# Mycogenic Nanomaterials: What Fungal Nanoparticles Promise and What Still Holds Them Back

**DOI:** 10.3390/jof12050366

**Published:** 2026-05-16

**Authors:** Kasun M. Thambugala, Sanduni Dabare, Asanthi Dhanusha, Imalka Munaweera, Dinushani A. Daranagama, Sukanya Haituk, Ratchadawan Cheewangkoon

**Affiliations:** 1 Genetics and Molecular Biology Unit, Faculty of Applied Sciences, University of Sri Jayewardenepura, Nugegoda 10250, Sri Lanka; asanthidhanusha@gmail.com; 2 Center for Plant Materials and Herbal Products Research, University of Sri Jayewardenepura, Nugegoda 10250, Sri Lanka; 3 Center for Biotechnology, Department of Zoology, University of Sri Jayewardenepura, Nugegoda 10250, Sri Lanka; 4 Department of Chemistry, Faculty of Applied Sciences, University of Sri Jayewardenepura, Gangodawila, Nugegoda 10250, Sri Lanka; dabaresanduni@gmail.com (S.D.); imalka@sjp.ac.lk (I.M.); 5 Department of Plant and Molecular Biology, Faculty of Science, University of Kelaniya, Kelaniya 11300, Sri Lanka; anupamad@kln.ac.lk; 6 Department of Entomology and Plant Pathology, Faculty of Agriculture, Chiang Mai University, Chiang Mai 50200, Thailand; sukanya.h@cmu.ac.th

**Keywords:** biocompatibility, fungal biosynthesis, green nanotechnology, bioprospecting, regulatory standardization

## Abstract

Mycogenic nanomaterials, nanoparticles (NPs) biosynthesized through fungal enzymatic and metabolic activity, have emerged as a compelling alternative to chemically synthesized nanomaterials, offering fundamental biocompatibility, green production conditions, and biologically functional surface coatings. Fungi, acting as natural “nanofactories,” harness reductases, oxidoreductases, secreted proteins, and secondary metabolites to reduce metal ions into stable NPs under ambient conditions, simultaneously capping the particles with biomolecules that enhance colloidal stability, biocompatibility, and secondary biological activity. Unlike previous reviews that have addressed either biosynthesis mechanisms or applications in isolation, this review uniquely adopts a structured “Promise vs. Barrier” framework across six interconnected thematic pillars, offering the first comprehensive critical synthesis that simultaneously maps mechanistic frontiers, biodiversity gaps, and translational barriers within mycogenic nanotechnology. The present review critically examines both the extraordinary promise and the persistent barriers facing mycogenic nanotechnology across biosynthetic mechanisms, fungal biodiversity, nanomaterial portfolio expansion, biomedical applications, environmental and agricultural utility, and industrial scalability. We highlight how emerging multiomics approaches, integrating transcriptomics, proteomics, and metabolomics, are beginning to decode the molecular blueprints of fungal NP synthesis, while acknowledging that mechanistic knowledge gaps, limited genetic toolkits for non-model fungi, and the absence of standardized protocols continue to impede progress. The fungal kingdom represents a vast, underexplored reservoir of nanofactory potential, with fewer than 1% of known species evaluated to date; strategic bioprospecting using genome mining and machine learning is beginning to unlock this diversity. Mycogenic NPs demonstrate broad-spectrum antimicrobial activity against multidrug-resistant pathogens, selective anticancer activity, biosensing capacity, and applications in wound healing, sustainable agriculture, environmental remediation, and smart food packaging. However, critical deficits persist in clinical validation, long-term toxicity data, manufacturing reproducibility, and regulatory clarity. The review concludes with a tiered roadmap, spanning immediate mechanistic priorities through to long-term synthetic biology and AI-integrated commercialization, and calls for coordinated international action on standardization, reference material development, and harmonized regulatory frameworks to bridge the gap between laboratory promise and real-world application.

## 1. Introduction

The merging of mycology and nanotechnology has given rise to a rapidly maturing field of mycogenic nanotechnology, where fungi serve as living bioreactors for the synthesis of functional nanomaterials [1,2]. Over the past two decades, a compelling body of evidence has established that fungi, from common soil saprophytes to rare marine extremophiles, possess an extraordinary capacity to convert dissolved metal ions into size-defined, crystalline NPs under ambient, aqueous conditions [3,4,5,6]. Unlike conventional physicochemical synthesis routes that demand elevated temperatures, hazardous reductants, and energy-intensive procedures, fungal biosynthesis proceeds through enzymatic reduction and metabolite-mediated stabilization, inherently aligning with the principles of green chemistry [7,8]. The resulting nanomaterials carry a biological “fingerprint”: a corona of fungal proteins, polysaccharides, and secondary metabolites that simultaneously stabilizes the particles and imparts secondary biological activities ranging from antimicrobial to anticancer effects [6,9,10].

Despite its impressive potential, mycogenic nanotechnology now faces significant challenges that mark a critical phase in its development. The scientific literature is rich with demonstrations of NP synthesis by individual fungal species, mainly *Aspergillus*, *Fusarium*, *Penicillium*, and *Trichoderma* spp. [11,12,13,14], yet the field has struggled to transition from proof-of-concept laboratory experiments to reproducible, scalable, and clinically viable products [15,16,17]. This paradox arises from a gathering of unresolved challenges spanning multiple dimensions: incomplete mechanistic understanding of fungal biosynthetic pathways; limited exploitation of the vast phylogenetic diversity of fungi; manufacturing and scale-up barriers; a critical deficit of standardized protocols and regulatory frameworks; and an emerging but as yet immature knowledge infrastructure for data sharing and computational optimization [15,18,19,20]. Notably, fewer than 1% of estimated fungal species have been evaluated for nanomaterial production, representing an extraordinary and largely untapped reservoir of biosynthetic diversity [21].

The global nanomaterials market is projected to exceed USD 290 billion by 2030, with biogenic nanomaterials increasingly valued for their reduced environmental footprint and functional advantages [22]. Antimicrobial resistance (AMR), declared by the World Health Organization (WHO) as one of the ten greatest public health threats of our time, is driving urgent demand for novel antimicrobial agents, a demand that mycogenic silver, copper, and zinc oxide NPs are well positioned to address [23,24]. Simultaneously, growing regulatory pressure on synthetic chemical processes and increasing consumer preference for bio-derived, sustainable materials are creating favorable conditions for mycogenic nanomaterial technologies [14,23,25]. Yet, the path from fungal broth to a commercial product remains fraught with unresolved scientific, engineering, and regulatory barriers.

Existing reviews of mycogenic nanomaterials have largely documented synthesis methods and applications by fungal species or NP type. This review takes a deliberately critical perspective, structured around a recurring “Promise vs. Barrier” framework that pairs each area of scientific opportunity with an honest appraisal of what impedes its realization. We examine the field across six thematic pillars: (1) biosynthetic mechanisms and multiomics approaches to decode them; (2) fungal biodiversity as an underexplored nanofactory library; (3) the expanding portfolio of fungal-derived nanomaterials and their functional properties; (4) biomedical applications spanning antimicrobial therapy, cancer theranostics, biosensing, and regenerative medicine; (5) environmental and agricultural applications; and (6) the scalability, regulatory, standardization, and knowledge infrastructure challenges that must be overcome for commercial translation. The review concludes with a prioritized roadmap that articulates short-, medium-, and long-term actions for the field, emphasizing the centrality of interdisciplinary collaboration, harmonized standards, and data-driven innovation. By mapping both the promise and the barriers with equal rigor, this review aims to provide a realistic yet optimistic guide for researchers, policymakers, and industry stakeholders, enabling targeted strategies to overcome translational bottlenecks and realize the full potential of mycogenic nanotechnology.

## 2. The Promise: Understanding Fungal Biosynthesis Mechanisms

Fungi can be simply described as natural agents to create the NPs. Over time, they have developed the ability to produce a wide range of enzymes, proteins, and other small molecules that can convert metal ions into stable and useful NPs [14,21]. In contrast to conventional physical and chemical synthesis methods, which frequently rely on elevated temperatures, high pressure, and potentially hazardous reducing agents, fungal biosynthesis proceeds under ambient conditions. The process is naturally aligned with green chemistry principles, utilising biologically derived reducing and stabilising molecules for NP formation [26].

Usually, fungal systems do more than simply reduce metal salts into nanoscale materials. The same biomolecules responsible for reduction often remain associated with the NP surface, acting as natural capping and stabilising agents [27]. This biologically mediated surface functionalisation can enhance colloidal stability and biocompatibility. According to Šebesta et al. [27], these same features are often challenging to reproduce through purely synthetic approaches. Thus, fungal NP synthesis represents not only an environmentally sustainable alternative, but also a biologically integrated strategy for generating functional nanomaterials with intrinsic surface chemistry.

### 2.1. What We Know: Established Biosynthetic Pathways

Through sophisticated biological mechanisms, fungi produce nanoparticles from metal ions. Microbial resistance strategies for cellular detoxification affect the solubility of inorganic and harmful ions by enzymatic reduction and nanostructure precipitation [28]. Microorganisms reduce metal ions to nanoparticles to withstand heavy metals [29]. Fungi produce a wide range of enzymes that catalyse the reduction process, and their redox potential and secondary metabolites may mediate electron-transfer pathways to produce stable nanoparticles [30]. Biosynthesis reduces metal ions to zero-valent form using enzymatic systems, NADH-dependent reductases, and other macromolecules [31].

**Figure 1:**
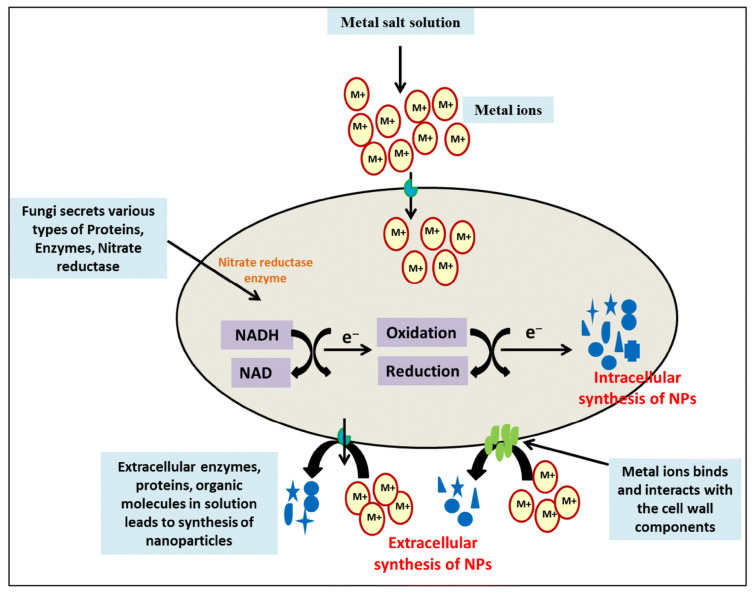
The possible mechanism of nanoparticle synthesis using fungi [15].

Intracellular and extracellular fungal nanoparticle production. For extracellular nanoparticle formation, microbial enzymes and proteins, bacterial or fungal cell wall components, or organic compounds in the culture media decrease metal ions. The carboxyl groups of microbial cell walls electrostatically attract metal ions, which pass into the cells and are reduced by intracellular proteins and cofactors. Oxidoreductases such as NADH-dependent nitrate reductase, NADPH-dependent sulfite reductase flavoprotein subunit α, cysteine desulfhydrase, and cellular transporters are common routes [16,32]. Enzymatic mechanisms, precipitation, complexation, peptide binding, and efflux pumps assist the cell remediate metal during biosynthesis, leading in a metal-resistant phenotype [33]. Fungal proteins, polysaccharides, and organic acids may discriminate crystal shapes and create lengthy spherical crystals [34]. Several bioactive macromolecules stabilize nanoparticle production thermodynamically [35,36]. Figure 1 shows the possible mechanism of nanoparticle synthesis using fungi.

#### 2.1.1. Enzymatic Reduction

The enzymatic reduction of metal ions in fungal nanoparticle biosynthesis is primarily driven by oxidoreductase enzymes that facilitate electron transfer processes [32]. The most well-characterized pathway involves NADH-dependent nitrate reductase, which has been extensively documented in *Fusarium oxysporum* and other fungal species [12,37,38,39]. This enzyme normally functions in nitrogen metabolism but can redirect its electron flow to reduce metal ions instead of nitrate, converting silver ions (Ag^+^) to metallic silver (Ag^0^) through electron transfer from NADH or NADPH [40,41].

Oxidoreductase enzymes represented another major enzymatic class. In *Fusarium oxysporum* secretomes, glyceraldehyde reductase and FAD-oxidoreductase were identified through proteomic analysis [42]. These redox proteins donated electrons to reduce silver ions (Ag^+^) to metallic silver (Ag^0^) [42] and generated reactive oxygen species [42]. Phenol-oxidizing enzymes, including laccases, tyrosinases, and manganese peroxidases, were isolated from Basidiomycetes and characterized for their role in metal recovery [43,44]. Species-specific enzymatic systems showed distinct localization patterns. In *Phanerochaete chrysosporium*, laccase dominated extracellular gold nanoparticle synthesis, while ligninase was responsible for intracellular nanoparticle formation on fungal mycelium [45]. Enzyme assays confirmed their role as both reducing and shape-directing agents [45]. The enzymatic reduction followed a characteristic two-step pathway for gold nanoparticles, where Au^3+^ was first reduced to Au^+^ and then to Au^0^ [46]. NADH/NADPH oxidoreductases facilitated this electron transfer process [2].

Different fungal species employ various specialized enzymes for nanoparticle synthesis. In *xylotrophic* fungi, phenol oxidase enzymes including laccase, Mn-peroxidases, and tyrosinases drive the reduction process [13,47,48]. *Saccharomyces cerevisiae* utilizes membrane-bound and cytosolic oxidoreductases along with extracellular β-glucan synthase [16]. Other important enzymes include sulfite reductase, hydrogenase, FAD-dependent glutathione reductase, and various ATPases that contribute to intracellular reduction processes [32,49,50].

The enzymatic reduction occurs through both extracellular and intracellular pathways. In extracellular synthesis, secreted reductases and proteins in the fungal filtrate reduce metal ions in the surrounding medium [39,51]. For intracellular synthesis, metal ions are first trapped by electrostatic interactions with cell wall components, then reduced by cytoplasmic enzymes and cell wall-associated reductases [41,43]. Small molecules under 3 kDa, including amino acids, cofactors, and glucose-based substances, also contribute to the enzymatic reduction process [43,52].

#### 2.1.2. Non-Enzymatic Metabolite-Mediated Reduction

Fungi use many non-enzymatic metabolites as reducing agents in nanoparticle production. Proteins, polysaccharides, flavonoids, alkaloids, phenolic acids, and other organic substances are produced during fungal development and stress reactions [41]. Metabolite reduction and stabilization promote nanoparticle formation and avoid aggregation [41].

Small molecules under 3 kDa play particularly important roles in the reduction process. These include amino acids, cofactors, and glucose-based substances that can effectively reduce metal ions through electron donation [43,52]. Cell wall sugars are especially significant contributors to metal ion reduction, working in concert with proteins and amino acids containing sulfhydryl (-SH) groups such as cysteine [41]. These cysteine-containing compounds undergo dehydrogenation reactions with metal salts, directly producing nanoparticles without enzymatic intervention [41]. Protein-based non-enzymatic mechanisms were documented in *Penicillium cyclopium*. FT-IR analysis revealed that saccharides and proteins on the mycelium surface participated in silver nanoparticle formation [53], with a specific polypeptide fraction of approximately 5000 Da identified as crucial for biomineralization [53]. Enzymatic synthesis was explicitly excluded in this system [53].

*Fusarium oxysporum*, naphthoquinones and anthraquinones functioned as electron shuttles [54], with specific compounds like 2-acetyl3,8-dihydroxy-6-methoxy anthraquinone identified through thin-layer chromatography [54]. These quinone groups enabled chemical reduction pathways through electron shuttle processes [54].

Polysaccharides represent another major class of non-enzymatic reducing agents. High molecular weight exopolysaccharides can act as both reducing and stabilizing agents, with their hydroxyl groups facilitating metal ion reduction while providing surface stabilization for the formed nanoparticles [55,56]. Carbohydrates such as glucose and fructose present in fungal biomass filtrates serve as effective reducing agents for nanoparticle biosynthesis [57,58].

Fungi create citric and oxalic acids, which reduce and stabilize nanoparticles [59]. Terpenoids and alkaloids may reduce or cap the process [57]. Non-enzymatic electron transport processes by phenolic compounds are crucial to metal salt bio-reduction [60].

Metabolomic analysis of *Sarocladium subulatum* revealed 40 fungal metabolites capable of reducing silver ions [61]. Carboxyl (COOH) and hydroxyl (OH) functional groups played pivotal roles in this reduction [61]. Molecular docking studies determined that these metabolites did not contribute to antibacterial activity, indicating their function was specific to metal ion reduction [61].

Additional non-protein reducing agents included phytochelatins synthesized by *Candida albicans* involving glutathione [46], and melanin secreted by *Yarrowia lipolytica* that reduced Au^3+^ to Au^0^ [46]. Aromatic and sulfurcontaining amino acids (tyrosine, tryptophan, cysteine, methionine) were implicated in gold nanoparticle reduction through Au-S bond formation [43]. The broader metabolite landscape included extracellular polymeric substances containing carboxyl and hydroxyl groups for metal biosorption [43], organic acids that solubilized metals [43], melanins with high metal sorption capacity [43], and amino acids acting as reducing and capping agents [43,62].

Non-enzymatic reduction generally uses specialized fungal biomolecules that produce stable nanoparticle bioconjugate systems. Lipid and amide groups help produce selenium nanoparticles, and several proteins attach to their surfaces to stabilize them [63,64]. Fungal synthesis techniques benefit from their dual mechanism approach, where the same metabolites decrease metal ions and stabilize nanoparticles [65].

### 2.2. What Holds Us Back: Mechanistic Knowledge Gaps

Despite these well-documented elements, critical knowledge gaps persist in our molecular understanding of fungal NP biosynthesis. We know that it works, but we often do not know exactly how at a molecular level. In this review article, we have identified several barriers that can hinder the application of knowledge.

• Barrier 1—Incomplete molecular understanding of species-specific pathways: While broad classes of enzymes and metabolites are known to participate, the exact molecular cascades in most fungi remain uncharacterised. There is significant species level and strain level variability in the enzymes expressed, their regulation and in how these biochemical pathways integrate with metal-ion stress responses. This strain specificity contributes to inconsistent NP yields and properties across studies, limiting the development of predictable, generalisable mechanistic models [66]. For example, the biosynthetic “blueprint” for *Aspergillus niger* is drastically different from that of *Fusarium oxysporum*. We currently lack a universal molecular model that explains why some species produce spherical particles while others produce prisms under the same conditions [67].
• Barrier 2—Unknown identity of specific capping biomolecules: Although proteins and polysaccharides are broadly identified as capping agents, the specific identities and functions of these molecules, including their binding modalities and influence on NP physicochemistry, are often not known in detail. While we recognize that “proteins” cap the particles, the specific identity and amino acid sequences of these biomolecules remain largely uncharacterized for most fungal species Šebesta et al. [27]. Without defining these biomolecules at the structural and functional level, rational control of NP size, shape, and surface functionality remains elusive. This lack of identity makes it impossible to standardize the biological “shell” of the NP [68].
• Barrier 3—Lack of genetic manipulation tools for optimisation: Many fungi that are prolific NP synthesizers lack well-developed genetic toolkits. The inability to perform targeted gene knockouts, overexpression, or pathway engineering hampers efforts to optimize biosynthetic flux towards desired NP traits [69]. This is especially true outside a few model species where CRISPR/Cas systems and curated genome assemblies are not yet standard protocols. While we can edit the genomes of model organisms like *Saccharomyces cerevisiae*, many high-performing “nanofactory” fungi are non-model organisms with few established CRISPR/Cas9 protocols [70]. This prevents us from “turning up the volume” on specific reductase genes to optimize yields.

### 2.3. The Promise of Multiomics Approaches

To pierce through these barriers, the field is moving toward multiomics, a systems-biology approach that examines the fungus’s entire biological response simultaneously. Integrating high-throughput omics technologies, such as transcriptomics, proteomics, and metabolomics holds immense promise for resolving the structural and mechanistic complexity of fungal NP biosynthesis.

Transcriptomics provides genome-wide expression profiles that reveal how fungi regulate genes involved in metal uptake, stress responses, and reductive metabolism under NP synthesis conditions [71]. By analyzing the mRNA levels, researchers can identify which genes are “switched on” the moment a fungus is exposed to a metal salt, revealing the genetic triggers for synthesis. By capturing dynamic transcript changes, researchers can pinpoint candidate pathways responsive to metal ions [72].

Proteomics expands this insight by identifying expressed proteins and post-translational modifications, critical for understanding active enzymes (e.g., reductases and oxidoreductases) and structural proteins that contribute to capping and stabilisation. This is the gold standard for identifying the enzymes involved. High-resolution mass spectrometry allows for the identification of the exact reductases and capping proteins present in the fungal secretome. Proteomic characterisation also reveals secreted proteins that directly participate in extracellular NP formation [20].

Beyond proteins, fungi produce secondary metabolites (alkaloids, flavonoids) that contribute to reduction. Metabolomic profiling identifies these small molecules, providing a complete map of the chemical environment [73]. Metabolomics sets gene and protein data by profiling the small molecules that serve as electron donors, redox mediators, and capping ligands. Metabolomic maps can identify flavonoids, phenolics, organic acids, and secondary metabolites that modulate NP nucleation and growth [74].

When these different data layers are integrated, we can begin to predict how a specific fungal strain will behave, enabling us to engineer NPs with specified size, shape, and surface chemistry. Crucially, integrative multiomics constructs a system that correlates gene regulation with enzymatic presence and metabolite production, enabling realistic design of fungal strains and culture conditions for tailored NP synthesis [27].

Multi-omics approaches offer insights into fungal nanoparticle biosynthesis by linking genetic potential to function and metabolism. For example, integrated transcriptomic-proteomic analyses identify upregulated reductases and stress pathways under metal exposure, revealing molecular triggers for nanoparticle formation. Combined transcriptomics and metabolomics map regulatory networks controlling fungal metabolism and secondary metabolites, showing how genes, enzymes, and metabolites act during biochemical transformations [75]. These approaches help identify key reductive enzymes like nitrate reductases, characterize capping biomolecules, and relate metabolite profiles to nanoparticle size, shape, and stability. They enable moving from simple observations to understanding mechanisms and designing fungal nanofactories [76].

### 2.4. What Still Holds This Back

Despite the promise of multiomics, several obstacles must be cleared during the transition from theory to practice.

• Barrier 1—Limited omics data for most fungal species: Current databases are heavily biased toward human and plant pathogens. Industrial-scale fungi used for nanomaterials often have poorly annotated genomes, making omics data difficult to interpret [3]. Comprehensive omics datasets remain concentrated in a few model organisms, with most NP-producing fungi lacking annotated genomes or sequencing resources. This scarcity stops comparative analyses and pathway reconstruction across diverse taxa.
• Barrier 2—Computational integration challenges: Merging a proteome (proteins) with a metabolome (chemicals) requires immense computational power and complex algorithms. The “big data” generated is often so noisy that key biosynthetic signals are lost. Multiomics integration requires sophisticated computational frameworks to align disparate data types (e.g., transcripts, proteins, metabolites) into coherent networks. Current analytical challenges include data standardisation, noise reduction, and functional annotation, especially in fungi with poorly characterised gene families [77].
• Barrier 3—Cost and technical expertise requirements: On the other hand, multi-omics approaches requires expensive sequencing and mass spectrometry equipment, as well as highly specialized bioinformaticians. For many research groups in developing economies, this financial and technical barrier remains the primary obstacle to moving beyond simple characterization [78]. High costs of sequencing, mass spectrometry, and data analysis infrastructure, coupled with the need for specialized expertise, limit the widespread adoption of multiomics in fungal NP research, particularly in low-resource settings [77].

Solving these bottlenecks is essential to bridge the gap between ‘omics’ theory and real-world application. It is the key to evolving mycogenic synthesis from a laboratory experiment into a reliable, mass-production technology for the next generation of nanomaterials.

Future research should develop integrated systems biology frameworks combining multi-omics data, computational modeling, and experimental validation. Advances in bioinformatics, like machine learning and network analysis, can improve dataset interpretation and identify key regulatory hubs [79]. Expanding fungal genome databases and refining annotation pipelines will enhance omics-based predictions [80]. Coupling multi-omics with functional genomics tools (e.g., CRISPR) and real-time techniques can bridge correlation and causation [79]. These strategies will shift from exploratory studies to rational engineering of fungal systems for scalable nanoparticle synthesis.

## 3. The Promise: Fungal Biodiversity as a Nanofactory Library

While early myconanotechnology relied on a handful of “lab favourites,” researchers are now realizing that the fungal kingdom is a vast, untapped library of organisms. Each fungal lineage carries a distinct metabolic signature, shaped by ecological adaptation, environmental pressures, and evolutionary history. This biochemical diversity translates directly into variation in reducing capacity, enzyme repertoire, secretome composition, and ultimately, NP characteristics.

### 3.1. The Promise of Diverse Fungal Sources

Historically, research has centred on well-characterised genera such as *Fusarium*, *Aspergillus*, and *Penicillium*, largely due to their ease of cultivation and established genetic knowledge. These classical models have demonstrated consistent ability to synthesise silver, gold, zinc oxide, and magnetite NPs, often through secreted reductases and protein-mediated stabilisation mechanisms [14,81]. While foundational, these genera represent only a narrow window into fungal diversity, akin to the tip of the iceberg.

Increasing attention is now being directed toward endophytic fungi, which reside asymptomatically within plant tissues. They have evolved unique metabolomes to survive in symbiosis; as a result, they frequently produce secondary metabolites influenced by host-microbe interactions, leading to distinctive redox chemistries and NP morphologies. Their metabolomic plasticity positions them as promising candidates for generating nanomaterials with novel surface functionalities and enhanced bioactivity [82]. Species such as *Talaromyces purpureogenus* are now being recognized for their ability to use extracellular pigments (like monascus-like azaphilones) as powerful reducing agents, creating highly stable silver NPs without the need for complex extraction steps [83,84,85].

Similarly, marine fungi offer compelling opportunities. Adapted to saline environments, osmotic stress, and fluctuating temperatures, marine-derived enzymes often display salt tolerance, thermostability, and altered catalytic efficiencies [86]. These traits may translate into robust NP synthesis under conditions that challenge terrestrial strains, broadening the operational window for biosynthetic nanotechnology. Even more intriguing are extremophilic fungi isolated from environments such as hot springs, hypersaline lakes, or arid ecosystems. These organisms harbour unconventional biochemical pathways shaped by environmental stress, potentially yielding unique NP morphologies or compositions [87,88]. Fungi adapted to high salinity or extreme temperatures offer enzymes that are naturally more stable than their terrestrial counterparts. For instance, Ali et al. [89] reported that marine-derived *Fusarium equiseti* has shown remarkable efficiency in creating monodisperse particles due to its adapted metabolic resilience. Recent reports on species within the genus *Talaromyces* highlight how relatively underexplored taxa can produce NPs with distinct size distributions and biological properties [90].

### 3.2. What Holds Us Back

Despite the immense promise of fungal biodiversity, several critical constraints limit its translation from the lab to the factory.

• Barrier 1—<1% of fungal species explored for nanoparticle synthesis: It is estimated that millions of fungal species exist globally, yet only a minute fraction have been evaluated for nanomaterial production. Currently, less than 1% of known fungal species have been tested for their “nanofactory” potential [1]. Current literature disproportionately focuses on a handful of genera, leaving most fungal phylogenetic space unexplored [91]. This imbalance restricts the application of the potential fungi in this arena.
• Barrier 2—Lack of standardized screening protocols: Every lab uses different growth media, temperatures, and pH levels. This makes it nearly impossible to compare results or guarantee. Comparative assessment across studies is often hindered by variability in metal precursor concentrations, incubation times, pH conditions, biomass preparation methods, and characterisation techniques [92,93]. Without harmonised protocols, reproducibility and cross-study benchmarking remain challenging, slowing systematic progress.
• Barrier 3—Difficulties in culturing rare/unculturable fungi: Many fungi, particularly endophytic, marine, or extremophilic species, require specialised growth conditions that are difficult to replicate in laboratory settings [94]. Some remain unculturable with current techniques, limiting access to their biosynthetic potential. The absence of culture-independent NP screening approaches further limits assessment.
• Barrier 4—Limited mycological expertise in nanotechnology labs: Nanotechnology-focused research groups may lack deep taxonomic and ecological knowledge of fungi, while mycology laboratories may not possess advanced nanomaterial characterisation infrastructure [94,95]. Most nanotechnology labs are staffed by physicists and chemists, while mycology labs focus on biology. This disciplinary gap can impede the development of rigorous strain identification, preservation, and optimisation strategies.

Therefore, these identified barriers highlight that the challenge is not a shortage of fungal potential, but rather a shortage of integrated exploration frameworks.

### 3.3. The Promise: Bioprospecting Strategies

To unlock fungal biodiversity as a systematic nanofactory resource, strategic and data-driven bioprospecting approaches are essential.

Modern fungal selection no longer starts in a Petri dish; rather, it starts in a database. By using genome mining, researchers can scan fungal DNA for Biosynthetic Gene Clusters that code for specific reductase enzymes or metal-binding proteins. Advances in fungal genomics enable the identification of genes encoding reductases, oxidoreductases, metal transporters, and secondary metabolite biosynthetic gene clusters. If a fungus has genetic blueprints for high nitrate reductase production, it is a prime candidate for silver NP synthesis, even before it is grown in the laboratory [96]. Likewise, mining genomic datasets can prioritise strains with theoretical NP-producing potential before experimental validation. Predictive annotation of secretome composition and redox-active enzymes offers a rational starting point for strain selection.

Miniaturised culture systems combined with spectroscopic and colorimetric assays allow rapid preliminary detection of NP formation. Automated screening platforms can evaluate multiple strains, metal precursors, and environmental conditions simultaneously, accelerating discovery while reducing resource expenditure [97].

The most exciting frontier is the use of Machine Learning (ML). By feeding algorithms data from previous successes and failures, we can build models that predict which fungal species will produce the best particles [66]. These models analyse variables such as protein secretion profiles and environmental origins to identify “winners,” effectively serving as a GPS for researchers navigating the vast landscape of fungal biodiversity. Integrating phylogenetic data, ecological origin, metabolomic profiles, and genomic features into computational frameworks can guide predictive selection of promising candidates [98]. In Golnaraghi-Ghomi et al. [98] researchers used artificial neural networks to model and optimize the eco-friendly production of zirconium NPs using common *Penicillium* fungi. ML models trained on known NP-producing fungi may identify patterns that link taxonomy or ecological niche to nanomaterial output. For example, by combining ML with NP-enhanced spectroscopy, researchers have developed a high-speed system for accurately identifying and measuring the fungal pathogen *Botryosphaeria dothidea* [99]. It is therefore evident that using data to predict outcomes allows us to move away from trial and error toward a more systematic and informed approach to discovery.

Recent advances demonstrate that ML and genome mining can shift fungal nanoparticle synthesis from empirical screening toward predictive and data-driven design. Machine learning techniques, including random forest (RF), support vector machines, and neural networks, have been widely applied in nanomaterial research to predict nanoparticle properties such as size, morphology, phase, and yield based on synthesis parameters. For example, RF models have shown high predictive performance in correlating experimental conditions (e.g., pH, precursor concentration, temperature) with nanoparticle characteristics, achieving strong accuracy in predicting particle phase and size and identifying key synthesis-driving variables [100]. Similarly, ML-assisted synthesis frameworks integrating Bayesian optimization and deep neural networks have been successfully used to optimize silver nanoparticle production by rapidly converging toward desired optical properties while reducing experimental trials [101]. More broadly, ML-guided approaches enable the identification of complex relationships between synthesis parameters and nanoparticle outcomes, significantly accelerating materials discovery and reducing trial-and-error experimentation [102,103]. These approaches can be extended to fungal systems by incorporating biological variables such as enzyme activity, secretome composition, and metabolic profiles into predictive models.

In parallel, genome mining provides a complementary strategy by identifying biosynthetic gene clusters encoding reductases, oxidoreductases, and secondary metabolite pathways involved in metal ion reduction and nanoparticle stabilization. When combined with ML, genome-derived features can be used to train predictive models that classify fungal strains based on their nanoparticle synthesis potential. For instance, RF and ensemble learning approaches have been successfully used to analyze nanoparticle-related datasets, identifying critical physicochemical and biological features that influence nanoparticle behavior and performance [104,105]. By integrating genomic data, enzyme profiles, and experimental synthesis outcomes, such models can prioritize high-performing fungal strains and predict optimal synthesis conditions. Collectively, these integrative strategies represent a transition toward AI-driven bioprospecting and rational optimization, enabling efficient discovery, screening, and engineering of fungal “nanofactories” for controlled and scalable nanoparticle biosynthesis.

In essence, the future of fungal nanotechnology lies not only in recognising biodiversity, but in systematically organising and interrogating it. By combining classical mycology with genomics, computational biology, and high-throughput nanomaterial analytics, fungal biodiversity can transition from a largely untapped reservoir into a structured and scalable nanofactory library.

## 4. The Promise: Expanding the Nanomaterial Portfolio

### 4.1. Diversity of Fungal-Derived Nanomaterials

Fungi are versatile in nanomaterial synthesis, producing metallic NP (silver (Ag), gold, copper, platinum), metal oxides (zinc oxide (ZnO), iron oxide, titanium dioxide), bimetallic systems, and specialized compounds like quantum dots and magnetite. This diversity stems from their enzymatic abilities and metabolic processes that reduce various metal precursors. The synthesis occurs through both live and dead fungal biomass, with specific fungal genera demonstrating preferences for particular nanomaterial types. For example, *Aspergillus fumigatus* and *Fusarium oxysporum* excel at Ag NP production, while *Verticillium* species can produce magnetite NP [106]. Thermophilic fungi, including *Thermomyces lanuginosus*, show particular capability for gold and iron NP synthesis [52,107]. Table 1 summarizes representative studies on fungal-mediated synthesis of metallic and metal oxide NPs, highlighting species diversity, synthesis strategies, optimized conditions, and physicochemical characteristics.

Mycogenic NPs vary in size, structure, and optical properties based on fungal species and nanomaterial type. Ag NPs ranged from 5 to 100 nm, with species-specific differences indicating fungal metabolism influences nucleation and growth. CeO_2_ NPs were smaller (5–20 nm), while Se and ZnO were larger (55–123 nm), reflecting material-dependent synthesis. Optical characterization revealed composition-specific peaks: Ag NPs exhibited SPR at 400–420 nm, while oxide or chalcogenide NPs were absorbed at lower wavelengths. Structural analyses indicated crystalline phases. A common feature was the use of biologically derived capping agents, such as proteins and functional groups (hydroxyl, amide, carboxyl), which improved stability and prevented aggregation.

The observed variations in NP synthesis primarily reflect inherent differences in fungal metabolic machinery and enzyme systems. The Naqvi et al. [111] comparative study directly demonstrated this principle by evaluating four *Aspergillus* species under identical conditions, finding that *A. fumigatus* exhibited a higher synthesis rate than *A. niger*, *A. flavus*, and *A. terreus* [111]. These differences likely stem from variations in the reductase enzymes and metabolites responsible for metal ion reduction. Similarly, *P*. *sanguineus* achieved a remarkably high yield of 98.9% [113], suggesting that white-rot fungi may possess particularly efficient reduction mechanisms. The chemical composition of the target nanomaterial fundamentally influences synthesis parameters and resulting particle characteristics. Ag NPs across multiple studies showed SPR peaks between 400 and 420 nm [110,111,113,116], reflecting the intrinsic optical properties of metallic Ag. In contrast, CeO_2_ NPs displayed absorption at 296 nm [112] and Se NPs at 260 nm [114], consistent with their distinct electronic structures. The larger particle sizes observed for Se (60.48–123.16 nm) [114] and ZnO (54.8–82.6 nm) [117] compared to most Ag NPs (typically 5–25 nm) [110,111,115,116] suggest that reduction kinetics and nucleation-growth dynamics differ substantially across nanomaterial types. The Rajput et al. [108] study provided critical mechanistic insights by systematically varying temperature and pH. Increasing temperature accelerated NP production but yielded smaller particles [108], indicating temperature-dependent nucleation rates where higher temperatures favor rapid nucleation overgrowth. pH variations predominantly affected morphology rather than size [108], suggesting that surface charge and reduction potential are pH sensitive. These findings explain why studies employing different synthesis temperatures produce NPs of varying sizes, even when using the same fungal genus.

A consistent finding across studies was the presence of biological capping agents that distinguished mycogenic NPs from chemically synthesized counterparts. The fungal-produced corona identified by Rajput et al. conferred superior colloidal stability in high-ionic-strength environments [108]. Gopinath et al. demonstrated that natural enzyme capping prevented agglomeration [112]. The high zeta potential values (−49.3 to −43.7 mV) observed for Se NPs [114] further confirm strong electrostatic stabilization. These organic layers, containing proteins [109], amino acids [110], and hydroxyl groups [112,114], represent a biological advantage that may enhance biocompatibility and functionality in applications.

### 4.2. Promise of Synergistic Functional Properties

**Figure 2:**
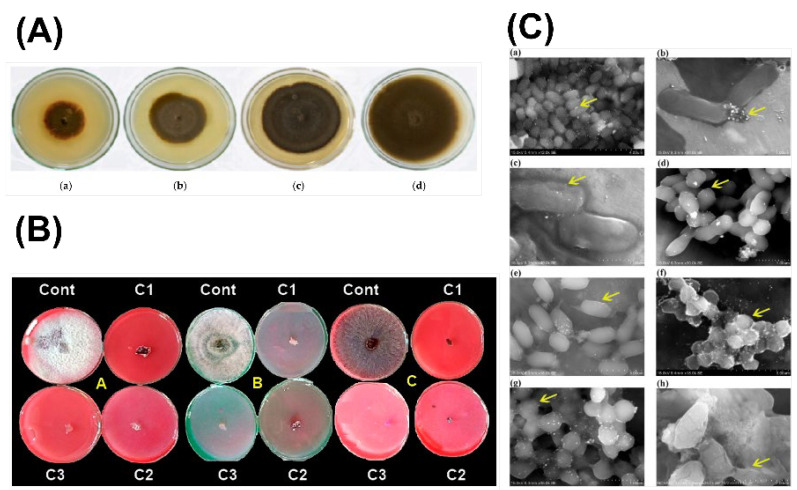
(**A**) In vitro antifungal activity of mycogenic SeNPs against *Pyricularia grisea* on PDA plates at different concentrations: (**a**) 200 ppm; (**b**) 100 ppm; (**c**) 50 ppm; (**d**) control [107], (**B**) The inhibitory effect of mycelia growth on F10 (A), Rs9 (B), M4 (C) on potato dextrose agar medium containing ZnONPs at concentrations: Control, (C1) 20, (C2) 40 and (C3) 100 μg/mL after 7 days [114], (**C**) Scanning electron micrographs of Ag NPs treated microbial cells at 6 h. (**a**) *E*. *coli *(4.00 μm scale); (**b**) *P*. *aeruginosa *(1.00 μm scale); (**c**) *K*. *pneumoniae *(1.00 μm scale); (**d**) *E*. *faecalis *(1.00 μm scale); (**e**) *B*. *subtilis *(3.00 μm scale); (**f**) methicillin-resistant *S*. *aureus *(3.00 μm scale); (**g**) *S*. *aureus *(1.00 μm scale); (**h**) *C*. *albicans *(2.00 μm scale). The arrow indicates the accumulation of NPs over the cell membrane and the breakage of the cell wall after treatment with Ag NPs [102].

Fungi not only form and stabilize NPs but also provide other biomolecules that have important properties in their application, such as the pharmaceutical effects of medicinal mushrooms or the antimicrobial effects of certain molecules that fungi like *Penicillium* species produce [27]. This synergy between the inorganic NP core and organic fungal capping represents an important avenue for future research [27]. The antimicrobial properties of mycogenic NPs demonstrate notable synergistic effects when combined with fungal extracts. Ag NPs synthesized using *Lactarius piperatus* mushroom extract showed synergistic antibacterial activity against various pathogenic microorganisms, while the mushroom extract reduced biofilm formation [118]. Similarly, Ag NPs synthesized using *Trichoderma harzianum* demonstrated enhanced biological activity due to fungal biomolecules that provide stability and contribute to biological effects [119,120]. Figure 2 illustrates the broad-spectrum antimicrobial potential of mycogenic NPs synthesized from different fungal systems.

Agricultural uses are another key area where mycogenic NPs show improved properties. ZnO NPs synthesised using *Trichoderma harzianum* as a biocontrol agent demonstrated novel fungicidal activity with complete inhibition of fungal growth against soil-borne plant pathogens, including *Fusarium* species, *Rhizoctonia solani*, and *Macrophomina phaseolina* [121]. Secondary metabolites released by biocontrol agents like *T*. *harzianum* operate as capping and reducing agents, helping to produce uniformly sized green and sustainable nanomaterials [119,120,121,122].

## 5. The Promise: Biomedical Applications

**Figure 3:**
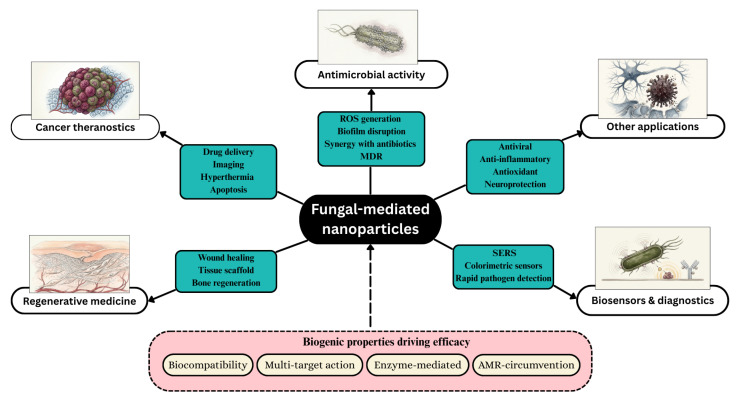
Biomedical applications of mycogenic NPs.

Mycogenic NPs serve as versatile nanomaterials with diverse biomedical applications. These include antimicrobial activity against pathogenic microorganisms, cancer theranostics for simultaneous diagnosis and therapy, regenerative medicine for tissue repair and wound healing, and biosensors and diagnostic systems for disease detection. Additional emerging biomedical applications are also supported by these nanomaterials. The diverse biomedical applications of fungal NPs are summarized in Figure 3.

### 5.1. Antimicrobial Warriors

Mycogenic NPs have emerged as promising antimicrobial agents due to their biogenic origin, structural complexity, and multifunctional mechanisms of action [123,124,125]. Unlike chemically synthesized nanomaterials, mycogenic NPs are produced through enzymatic reduction and stabilization processes mediated by fungal metabolites, including proteins, polysaccharides, phenolics, and reductase enzymes [125]. These biomolecules confer enhanced biocompatibility, improved stability, and intrinsic biological functionality. Consequently, mycogenic NPs are increasingly recognized as potent antimicrobial tools capable of addressing the growing global threat of AMR [126,127], particularly multidrug-resistant (MDR) pathogens and biofilm-associated infections [124,128].

One of the most significant advantages of mycogenic NPs lies in their ability to overcome biofilm-associated antimicrobial resistance. Biofilms, particularly those formed by fungal pathogens such as *Candida albicans*, exhibit high resistance to conventional antimicrobial agents due to the presence of a dense extracellular polymeric matrix [125]. This matrix restricts drug penetration and facilitates microbial persistence. Biogenic silver NPs synthesized using fungal systems have demonstrated the ability to penetrate biofilms, disrupt extracellular matrix integrity, and significantly reduce biofilm biomass, in some cases exceeding 70% reduction [125]. This disruption enhances microbial susceptibility and prevents further biofilm maturation.

Mycogenic NPs also exhibit synergistic interactions with conventional antimicrobial agents, including antibiotics and antifungal drugs such as ampicillin, kanamycin, and fluconazole [123,124,129]. This synergism enhances drug uptake, disrupts microbial defense mechanisms, and restores the effectiveness of antimicrobial agents to which pathogens have developed resistance [21]. Such combinatorial approaches offer a promising strategy for revitalizing existing antimicrobial therapies and mitigating resistance development [124].

The antimicrobial efficacy of mycogenic NPs is largely attributed to their multi-targeted mechanisms of action [123,124]. These include the generation of intracellular reactive oxygen species (ROS), disruption of microbial cell membranes, interference with metabolic pathways, and damage to nucleic acids and proteins. This multi-modal mode of action reduces the likelihood of resistance development compared to conventional antibiotics that typically target a single cellular pathway [123,124,130].

Extensive in vitro studies have demonstrated the broad-spectrum antimicrobial activity of mycogenic NPs against clinically relevant bacterial and fungal pathogens [15]. Silver NPs synthesized using fungal species such as *Aspergillus clavatus* and *Agaricus bisporus* have shown potent activity against methicillin-resistant *Staphylococcus aureus* (MRSA) [15,124,131], a major cause of hospital-acquired infections These NPs disrupt bacterial membrane integrity, induce oxidative stress, and inhibit essential cellular processes [124].

Similarly, mycosynthesized silver and copper NPs exhibit strong inhibitory activity against Gram-negative bacteria such as *Escherichia coli* [15,21,131]. These NPs compromise membrane permeability, disrupt respiratory enzyme systems, and induce oxidative damage. Mycogenic NPs also demonstrate significant antifungal activity, particularly against *Candida* species [21,125]. Biogenic silver NPs inhibit the yeast-to-hyphal transition in *Candida albicans*, a critical virulence factor required for tissue invasion and pathogenicity [21]. By preventing hyphal formation, mycogenic NPs effectively reduce fungal virulence and limit infection progression [125].

However, significant challenges remain, including limited clinical validation, incomplete mechanistic understanding, manufacturing variability, and regulatory uncertainty [125]. Addressing these barriers through standardized synthesis protocols, comprehensive safety evaluation, and harmonized regulatory frameworks will be essential to facilitate the safe and effective clinical translation of mycogenic NP-based antimicrobial therapies.

### 5.2. Cancer Theranostics

Cancer theranostics represents an emerging paradigm that integrates diagnosis and therapy into a unified nanoscale platform. Mycogenic NPs, along with other biogenic nanomaterials, have gained considerable attention as multifunctional systems capable of targeted drug delivery, imaging, and therapeutic intervention [130,132]. Their biogenic origin confers unique physicochemical and biological properties, including enhanced biocompatibility, intrinsic functionalization, and reduced toxicity compared to chemically synthesized counterparts [15]. These characteristics position mycogenic NPs as promising candidates for next-generation cancer theranostic applications.

One of the primary advantages of mycogenic NPs in cancer theranostics is their capacity for targeted drug delivery [15,21,132]. NPs can function as nanoscale carriers capable of encapsulating chemotherapeutic agents such as doxorubicin, paclitaxel, and methotrexate [15], thereby improving drug solubility, stability, and controlled release. Fungal systems serve as efficient biological nanofactories capable of synthesizing metallic NPs, liposomes, and magnetic nanostructures functionalized with biomolecules that facilitate cellular uptake and tumor targeting [15]. This targeted delivery approach improves therapeutic efficacy and reduces adverse side effects commonly associated with conventional chemotherapy.

In medical imaging, mycogenic NPs are also being explored for diagnostic purposes. Gold and silver NPs enhance contrast in electron microscopy and labeling, while cadmium telluride quantum dots and magnetic iron oxide NPs show promise as fluorescent markers and MRI contrast agents for detecting cancer cells, including those in the liver [21]. Beyond drug delivery and imaging, NPs also enable advanced therapies such as hyperthermia, where magnetic NPs generate localized heat under external magnetic fields to destroy tumor cells, and anti-angiogenesis, while blocking the formation of new blood vessels that tumors need for growth and survival [133].

Numerous in vitro studies have demonstrated the anticancer potential of mycogenic NPsagainst various cancer cell lines, including human lung carcinoma and breast cancer models [15]. For example, silver NPs synthesized using fungal species such as *Talaromyces purpureogenus* have been shown to induce apoptosis in human lung carcinoma cells while exhibiting minimal cytotoxicity toward normal fibroblast cells. This selective cytotoxicity is attributed to NP-induced oxidative stress, mitochondrial dysfunction, and activation of programmed cell death pathways [134].

Similarly, gold NPs synthesized using medicinal fungi such as *Inonotus obliquus* exhibit favorable properties for photothermal and combinational chemo-photothermal therapy [135]. Palladium NPs and other biogenic metallic nanostructures also demonstrate multifunctional therapeutic capabilities, including tumor cell destruction through thermal and oxidative mechanisms [55].

Despite promising experimental outcomes, several critical challenges limit the clinical implementation of mycogenic NPs in cancer theranostics. These critical challenges include insufficient pharmacokinetic data, unclear biodistribution, targeting limitations, tumor penetration barriers, and long-term safety concerns. Addressing these challenges through rigorous pharmacological evaluation, improved NP functionalization strategies, and comprehensive safety assessment will be essential to facilitate the clinical translation of fungal-mediated nanotheranostic systems and unlock their full potential in precision oncology.

### 5.3. Biosensors and Diagnostics

Mycogenic NPs and other biogenic NPs are gaining attention in biosensors and diagnostics because of their potential to support rapid, sensitive, and selective point-of-care testing [136]. These nano-enabled systems can significantly shorten pathogen detection times, reducing identification of organisms such as *Salmonella* or Hepatitis C from several days to only a few hours [136]. NPs serve as optical receptors in colorimetric sensors for detecting toxic substances like mercury ions, and they enhance analytical performance in Surface-Enhanced Raman Scattering (SERS) platforms, enabling ultrasensitive detection of proteins and multiple antimicrobial-resistant pathogens [137].

Despite this promise, several barriers limit their broader adoption [21]. Reproducibility remains a major challenge, especially in complex real-world biological or environmental samples [138]. Interlaboratory comparisons show substantial variability, highlighting sensitivity gaps compared with established synthetic standards [138]. In addition, many biogenic nanomaterials are inherently unstable, undergoing aggregation or degradation over time, which affects shelf-life [138]. Regulatory uncertainty further complicates clinical translation due to the absence of harmonized global definitions and standardized nomenclature [138].

### 5.4. Regenerative Medicine

In regenerative medicine, mycogenic NPs show strong potential in wound healing, tissue engineering, and bone regeneration [21]. Mycosynthesized silver NPs have demonstrated enhanced cell proliferation and significant wound healing activity in vitro scratch assays [134]. There are already clinical precedents for silver-based materials, including antimicrobial dressings for chronic wounds and silver-loaded bone cement used in joint replacement procedures to reduce infection risk [123].

However, progress toward broader clinical use is slowed by a critical shortage of in vivo studies and limited long-term toxicity data in human populations. Stable integration of NPs into biomaterial scaffolds for complex tissue regeneration also remains technically challenging and requires more targeted analytical testing [21]. Concerns about biocompatibility, bio-persistence, and bioaccumulation persist, as some cellular functions do not fully return to baseline even days after exposure, raising questions about potential long-term effects [130].

### 5.5. Other Medical Applications

Beyond diagnostics and regenerative medicine, fungal NPs are being explored for antioxidant, anti-inflammatory, antiviral, and neuroprotective applications [130]. Many fungal-derived NPs, particularly those capped with bioactive metabolites, demonstrate inherent antioxidant and anti-inflammatory properties [139]. Silver NPs synthesized from *Aspergillus niger* have also shown thrombolytic and anticoagulant activity [136]. Biogenic silver NPs have demonstrated antiviral effects against viruses such as HIV-1 and Hepatitis B by interfering with viral replication in a size-dependent manner [140]. In the context of neurodegenerative diseases, nanobiotechnology is emerging as a promising research area, with NPs being investigated for neurological applications and combination devices [136]. While their ability to cross the blood–brain barrier offers therapeutic opportunities, it also raises important safety considerations related to possible long-term neuronal effects [124].

## 6. The Promise: Environmental and Agricultural Applications

In this section we examine the practical applications of mycogenic nanomaterials beyond the laboratory, focusing on how these biological “nanofactories” can address global crises in pollution, food security, and agricultural sustainability.

### 6.1. Environmental Remediation

Mycogenic NPs offer a high-efficiency solution for nanobioremediation, particularly in the removal of heavy metals such as lead, chromium, and cadmium and the degradation of synthetic dyes from industrial effluents [15,141,142]. Fungal-derived NPs, especially silver (AgNPs), iron oxide (Fe_3_O_4_), zinc oxide (ZnO), and gold NPs exhibit high surface area, reactive surface chemistry, and catalytic potential, enabling efficient adsorption and transformation of pollutants [20]. Their biologically derived capping layers can further enhance colloidal stability and interaction with contaminants. Fungal-derived zero-valent iron (nZVI) and silver NPs (AgNPs) are particularly valued for their catalytic properties in breaking down organic contaminants [142].

In wastewater treatment, mycogenic NPs have demonstrated strong affinity for heavy metals such as lead (Pb^2+^), cadmium (Cd^2+^), chromium (Cr^6+^), and arsenic (As^3+^), functioning through adsorption, redox transformation, or catalytic reduction mechanisms. Similarly, dye-contaminated effluents from textile industries can be treated via NP-mediated photocatalytic or reductive degradation pathways, reducing color intensity and toxicity under mild conditions. These applications align closely with green chemistry principles, as fungal synthesis avoids harsh chemical reducing agents and energy-intensive procedures [143].

Filamentous fungi secrete powerful extracellular enzymes, including laccases and peroxidases, which facilitate the adsorption and catalytic degradation of toxic compounds [142]. For example, Penicillium and *Aspergillus* species have demonstrated near-total decolorization of complex azo dyes by transforming them into safer, non-colored structures [142]. Ali et al., 2024 [89] have shown that fungal-synthesised iron oxide NPs can facilitate magnetic recovery after heavy metal adsorption, improving separation efficiency. Likewise, ZnO and AgNPs biosynthesised using fungal filtrates have demonstrated catalytic degradation of azo dyes through ROS generation under light exposure.

Collectively, these findings indicate that mycogenic NPs are not merely laboratory curiosities but active catalytic and adsorptive agents with measurable environmental performance. Table 2 summarizes recent research-based studies on the application of mycogenic nanomaterials in environmental remediation, highlighting the diversity of fungal species, nanoparticle types, and targeted pollutants.

### 6.2. Sustainable Agriculture

Mycogenic NPs are being explored as nano-fertilisers, nano-pesticides, and plant growth-promoting agents. The development of “smart” fertilizers and pesticides, where NPs act as carriers for nutrients or bioactive compounds, promises to revolutionize crop yields while reducing chemical runoff [14]. Their small size enables enhanced nutrient solubility, controlled release, and improved foliar or root uptake efficiency. Nevertheless, these materials can improve nutrient uptake, enhance water retention, and strengthen plant immune responses [150].

Nano-fertilisers derived from fungal-mediated synthesis can enhance micronutrient delivery (e.g., Zn, Fe), reducing application rates while improving plant growth metrics. Similarly, AgNPs and ZnO NPs exhibit antimicrobial properties against phytopathogens, offering alternatives to synthetic agrochemicals [20,151]. Some fungal-derived NPs have also demonstrated elicitor-like activity, stimulating plant defense pathways and stress tolerance. Recent studies on endophytic fungi show they can synthesize nanomaterials that boost plant health through nitrogen fixation and siderophore production without harmful pathological effects [14]. These “bio-nanofertilizers” have been shown to maintain crop productivity even under abiotic stresses like drought or high salinity [150].

Experimental studies report improved seed germination rates, biomass accumulation, chlorophyll content, and yield parameters following NP-based nutrient supplementation. Additionally, antifungal and antibacterial activity of mycogenic NPs against plant pathogens has been widely documented, suggesting their dual role as nutrient carriers and protective agents [152].

These findings suggest that fungal nanomaterials could reduce chemical pesticide dependency while improving nutrient efficiency—key goals for climate-resilient agriculture.

### 6.3. Smart Food Packaging

Fungal-derived NPs are increasingly investigated in smart food packaging systems. Mycogenic NPs can be integrated into “bioactive” packaging to create antimicrobial films that inhibit the growth of food-borne pathogens like *Aspergillus* or *Penicillium* species [89]. Their antimicrobial properties can inhibit spoilage organisms, extending shelf life and reducing post-harvest losses. Incorporation of AgNPs or ZnO NPs into biodegradable polymer matrices has shown promise in suppressing bacterial growth on food surfaces [153].

Beyond antimicrobial protection, nanosensors integrated into packaging materials can function as freshness indicators by detecting pH shifts, gas emissions (e.g., ammonia), or microbial metabolites [154]. These materials can also serve as freshness indicators, changing color to signal when food has begun to spoil. Such systems align with sustainable packaging strategies when combined with biodegradable biopolymers and green-synthesised NPs [155].

## 7. The Safety Question: Promise vs. Precaution

### 7.1. The Promise of Biocompatibility

Mycogenic NPs and other biogenic NPs (synthesized using biological systems such as bacteria, algae, plants) are widely regarded as safer alternatives to conventional chemically synthesized nanomaterials because their green synthesis avoids toxic reducing agents and hazardous chemical residues [125,130,156]. A key advantage of these NPs is their natural biomolecule capping, which consists of fungal-derived proteins, enzymes, and metabolites [139]. This coating provides electrosteric stability, enhances biological activity, and improves compatibility with living systems by promoting safer biological interactions [55,139]. Unlike synthetic chemical coatings that may persist in the environment, these organic biomolecules can degrade naturally [139]. As a result, many studies have shown that biogenic silver NPs exhibit lower cytotoxicity toward mammalian cells compared to chemically synthesized NPs or commercial equivalents [55,139].

### 7.2. What We Know About Safety

Current safety knowledge is primarily derived from in vitro cytotoxicity studies and limited in vivo animal investigations [124]. In vitro studies using cell lines such as HaCat, 3T3, and A549 demonstrate that NP toxicity is influenced by several factors, including dose, particle size, surface characteristics, and the fungal species involved in synthesis [139]. Importantly, several studies report selective toxicity, in which NPs effectively inhibit pathogens or cancer cells while maintaining minimal toxicity toward healthy cells at therapeutic concentrations [139]. Animal studies suggest that mycogenic NPs can be systemically distributed and tolerated at low doses [124,130]. However, at higher concentrations, they may accumulate in organs such as the liver, kidneys, and lungs, potentially triggering inflammation and affecting normal organ function [124,130].

### 7.3. What Critically Holds This Back

Despite these promising findings, the clinical and regulatory acceptance of mycogenic NPs remains limited, constrained by several interconnected challenges that highlights the gap between current experimental evidence and the standards required for safe clinical translation and regulatory approval.

• Barrier 1—Lack of Long-Term Toxicity Studies: One of the most critical limitations in the safety evaluation of mycogenic NPs is the absence of comprehensive long-term toxicity data [21,123,156]. The majority of the existing safety studies are confined to or sub-acute exposure assessment over relatively short experimental timeframes (≤28 days), providing insufficient insight into the consequences of prolonged or repeated exposure. Fundamental questions regarding chronic toxicity, tissue-specific bioaccumulation, intracellular persistence, and the potential for delayed adverse effects in organs such as the liver, kidneys, and lungs remain largely unresolved. This deficiency is further compounded by the near-total absence of clinical trial data systematically evaluating the safety, pharmacokinetics, and therapeutic efficacy of mycogenic FNP-based formulations in human subjects. In the absence of Phase I–III clinical studies, it is not possible to establish definitive conclusions regarding therapeutic dosing, treatment efficacy, adverse effect profiles, and long-term safety. As a result, regulatory approval remains unattainable under current evidence-based standards. Collectively, the absence of both longitudinal preclinical toxicological data and structured clinical validation represents a fundamental and interdependent evidentiary gap. Addressing this gap is essential for accurately defining the safety profile of mycogenic FNPs and for enabling their responsible and effective translation into clinical applications.
• Barrier 2—Unknown Effects on Human Microbiome: The potential impact of mycogenic NPs on the human microbiome represents an underexplored yet increasingly recognized safety concern. Emerging evidence suggests that NP exposure may disrupt epithelial tissues and microbial balance in the gastrointestinal tract [130], with potential downstream effects on immune regulation, metabolism, and systemic health. Given the central role of the gut microbiome in maintaining physiological homeostasis, even modest perturbations could have significant health implications. However, mechanistic studies that systematically characterize the nature, extent, and reversibility of mycogenic NPs-microbiome interactions remain scarce, representing a critical gap in the current safety literature.
• Barrier 3—Immunogenicity Not Well Characterized: The immunogenic potential of mycogenic NPs represents a significant and incompletely characterized safety concern. Upon systemic administration, NPs may be recognized as foreign entities by the immune system, potentially triggering inflammatory or immune responses [123,130]. In addition, the biological corona of fungal-derived proteins, polysaccharides, and other metabolites that naturally envelop mycogenic NPs may further modulate immune recognition in ways that are difficult to predict or standardize across formulations. Consequently, comprehensive immunological profiling is essential but remains insufficiently addressed in the existing literature, limiting confidence in the immunological safety of these nanomaterials.
• Barrier 4—Environmental Ecotoxicology Data Scarce: The environmental safety of mycogenic NPs remains poorly understood. Following their release into natural ecosystems through agricultural application, industrial discharge, or improper disposal, NPs may persist, transform, and affect non-target organisms [21]. Key risks include bioaccumulation in food chains, disruption of soil microbial communities, and toxicity to aquatic life. However, most formulations lack systematic study. This gap highlights the need for standardized environmental risk assessments before their widespread use in agriculture or bioremediation.
• Barrier 5—No Standardised Testing Protocols: The absence of standardized safety testing protocols limits the comparability, reproducibility, and regulatory utility of existing toxicological data for mycogenic NPs t [136]. Across the current literature, toxicity assessments are conducted using heterogeneous cell lines, variable exposure conditions (concentrations and durations), inconsistent nanoparticle characterization standards, and divergent endpoint measurements, leading to inconsistent and sometimes conflicting results. This makes cross-study comparison and risk assessment difficult. The development and adoption of harmonized, internationally recognized testing protocols specifically designed for biogenic nanomaterials is therefore an essential prerequisite for generating the consistent, high-quality safety data required to support regulatory review and clinical advancement.
• Barrier 6—Protein Corona Effects Poorly Understood: When mycogenic NPs enter biological fluids, they rapidly form a protein corona that alters their surface properties, behavior, and toxicity compared to their original state. This layer can affect size, stability, cellular uptake, biodistribution, and immune interactions in ways that are difficulty to predict from in vitro studies. Its composition varies depending on the biological fluid, nanoparticle properties, and biological context, adding complexity to safety evaluation and therapeutic design [130]. Despite its importance, the protein corona of mycogenic NPs remains poorly understood, particularly regarding its impact on in vivo safety and efficacy.
• Barrier 7—Reproductive and Developmental Toxicity Unknown: The reproductive and developmental toxicity of mycogenic NPs remains largely unstudied. Since some nanoparticles can cross barriers like the placenta and blood-testis barrier, there are concerns about effects on fertility, embryonic development, and fetal growth. However, no systematic studies exist for mycogenic NPs, leaving risks such as teratogenicity and long-term effects unknown [136]. This gap highlights the need for dedicated preclinical research before their use in reproductive-age or pregnant populations.
• Barrier 8—Occupational Exposure Guidelines Absent: The lack of occupational safety guidelines for handling mycogenic NPs is a significant concern [136]. Workers engaged in large-scale fungal NP synthesis may be exposed to aerosolized nanoparticles and fungal byproducts through inhalation, skin contact, or ingestion, yet no specific exposure limits or safety standards exist. Current guidelines for nanomaterials or biological agents are insufficient for these hybrid materials. Developing dedicated safety protocols, supported by inhalation and dermal studies, is essential as production scales up.

### 7.4. The Way Forward

To address these challenges, future progress will depend on developing standardized nanotoxicology protocols that ensure reliable and reproducible safety evaluations [136]. The use of certified reference materials and validated measurement standards will help improve consistency across laboratories worldwide [138]. Long-term environmental monitoring will also be essential to understand ecological impacts and NP persistence [136]. Importantly, the adoption of Safe-by-Design principles will allow researchers to integrate safety considerations into NP development from the earliest stages, balancing therapeutic effectiveness with minimal risk [136]. These strategies are essential for closing the regulatory gap and enabling the safe clinical and industrial application of FNP technologies.

## 8. The Scalability Challenge: From Bench to Market

### 8.1. The Promise of Industrial-Scale Production

Fungi are ideal for large-scale nanomaterial production because they secrete extracellular enzymes, offering advantages over bacterial fermentation, which requires extra steps to clarify broth [4]. Their high capacity, surface area, and mycelial growth make fungi efficient biosynthetic agents [122]. Fungi are ideal for industrial nanomaterial synthesis due to their advantages, including easy scaling, cost-effectiveness, efficient handling, and large surface area of mycelia [155,157]. Their metal tolerance and ability to bioaccumulate metals, along with producing extracellular enzymes, enable the construction of diverse enzymes for nanomaterials [155]. Rapid growth facilitates maintenance in labs or industry, and using biomass for nanomaterials offers economic and sustainable benefits. Despite promising characteristics, a key challenge is translating lab protocols to an industrial scale. Many protocols remain unproven outside the lab, creating a gap for standardization and scale-up [158]. For mycogenic nanomaterials to be industrially viable, they must complement existing methods or offer a competitive, sustainable alternative [158].

### 8.2. Major Barriers to Industrial Translation

The transition from laboratory-scale mycogenic nanomaterial production to industrial application faces several critical barriers that must be addressed for commercial viability. Most published protocols remain laboratory curiosities unproven beyond the bench, creating a gap in scaling small procedures to standardized processes [158]. Only 1% of microbial nanotech is commercialized, with most metal ion-to-NP yields uninvestigated or poorly analysed [159,160]. Scaling up is complex and costly, requiring process optimization for industrial use [14,151,161]. Maintaining consistent NP size and shape during scale-up is challenging, as variations can affect product quality and function [14,162]. Uncontrolled growth conditions, nutrients, and fungal metabolism lead to poor NP uniformity and inconsistent final products [40,161]. Mycelium-based materials are limited to small prototypes due to a lack of standardization in production and characterization [163]. Production processes are labor-intensive, involving multiple steps and careful monitoring of parameters [164]. Current studies are mostly lab-based, with production hindered by low yields, high enzyme needs, and costly downstream processing [165]. Growing large mycelium volumes poses logistical challenges, especially on-site [166]. NP toxicity limits industrial transfer and requires further study [151]. Regulatory barriers arise from limited data on in vivo safety, toxicity, and environmental impact [40]. Understanding molecular mechanisms and developing risk assessments are crucial before commercial use [167]. High costs hinder wider adoption, with few companies achieving mass production due to manufacturing expenses, variability, and limited regulation [163,168]. Most studies are limited to in vitro experiments and face issues like batch inconsistencies, unclear toxicity limits, and lacking regulatory standards [168,169,170].

### 8.3. Emerging Solutions and Future Opportunities

The field is developing solutions to overcome barriers to industrial-scale mycogenic nanomaterials production. Advanced bioreactor systems are key technological breakthroughs for maintaining consistent conditions. Specialized bioreactors help optimize growth and substrate use, increase yields, and improve NP quality through efficient downstream processing, making large-scale, cost-effective production feasible [14,162]. Future research should focus on genetically engineering high-yield microbial strains, implementing bioreactor systems, and developing standardized isolation protocols [40]. Automation and AI (artificial intelligence) are transforming the production of fungal materials by enhancing efficiency and scalability. AI algorithms optimize conditions like temperature, humidity, and nutrients in real-time, reducing labour and variability [163,164,171,172]. ML predicts growth patterns and yields from historical data. Economic sustainability is addressed through innovative raw materials and production methods. Studies show low-cost agricultural and industrial waste as alternatives reduce costs and improve sustainability [173,174,175]. Advanced techniques like electrospinning and 3D printing enable customized fungal materials, broadening industry applications [173]. Mycelium composites are the most advanced fungal-based technology, with companies like Ecovative producing molded foams from mycelium and agricultural residues, validated at industrial scale, including by Dell and IKEA [176]. However, the field varies in maturity, with some technologies at commercial scale and others still at pilot or lab stages, showing that functionality alone does not ensure adoption [163,176]. Future research should focus on overcoming technical and regulatory hurdles using novel fermentation strategies and advanced bioreactors for scalable production [173]. It should also aim to establish structure-function relationships, develop green extraction methods, and expand fungal sources via synthetic biology and metabolic engineering [165]. The potential of fungal cultures in NP production and the lack of knowledge in myco-nanosynthesis highlight the need for detailed studies to optimize and scale up industrial biosynthesis [48].

## 9. The Regulatory and Standardization Gap

The clinical translation of fungal NPs is limited by regulatory frameworks originally developed for conventional chemical drugs, which have not been systematically updated to address the unique characteristics of biogenically synthesised nanomaterials.

### 9.1. The Promise of Regulatory Clarity

A fundamental prerequisite for any microorganism considered for nanobiotechnological application is Generally Recognized as Safe (GRAS) status, and notably, fungal species of relevance to NP synthesis consistently satisfy this designation [21]. Several fungal genera commonly employed in NP biosynthesis, including specific strains of Aspergillus (notably *A*. *oryzae* and *A*. *niger*) and *Trichoderma* (notably *T*. *reesei*), are formally classified as GRAS by the Food and Drug Administration (FDA) and are established in commercial food sector use [13,130], providing a well-characterised toxicological foundation that reduces uncertainty in safety evaluation.

This inherent biosafety foundation positions mycogenic NPs derived from GRAS-designated strains favourably within existing regulatory traditions for biological products. Materials derived from GRAS organisms may, in certain jurisdictions, qualify for streamlined or abbreviated evaluation pathways, particularly when the biological synthesis process does not introduce hazardous chemical residues [138]. This “safe-by-design” characteristic aligns with regulatory priorities emphasizing the reduction in toxicological risk at the material design stage and may simplify aspects of risk assessment related to chemical toxicity and residual contaminants [138].

In addition, regulatory agencies such as the U.S. FDA already have extensive experience evaluating fungal-derived biological products, including recombinant proteins, enzymes, and vaccines produced through fungal fermentation systems [177]. These existing regulatory pathways include well-defined Chemistry, Manufacturing, and Controls (CMC) requirements governing product consistency, sterility, impurity control, and manufacturing reproducibility. If mycogenic NPs are classified within biological product or combination product frameworks, these established regulatory mechanisms could provide a structured pathway for evaluation, rather than requiring entirely new regulatory paradigms. This existing institutional familiarity with fungal-derived biologics represents a significant regulatory advantage compared to novel synthetic nanomaterials lacking biological precedents.

However, biogenic origin does not exempt mycogenic NPs from comprehensive regulatory scrutiny. NP-specific characteristics, including size-dependent biological interactions, biodistribution, persistence, and immunogenic potential, must still be evaluated independently of the safety profile of the source organism. Regulatory acceptance therefore depends not only on biological origin but also on rigorous characterisation, reproducibility, and demonstrated safety of the final NP product [136,138].

### 9.2. What Holds This Back: The Regulatory Void

Fungal-mediated nanomaterials represent a rapidly advancing class of biologically derived nanostructures with applications in medicine, agriculture, environmental remediation, and biotechnology. Despite their promise, regulatory frameworks have not evolved at a pace sufficient to address their unique biological origin, structural complexity, and manufacturing variability [136]. Several key regulatory limitations constrain their safe standardization, commercialization, and clinical translation.

• Barrier 1—No Specific Regulations for Biosynthesised Nanomaterials: Major regulatory agencies such as the U.S. FDA have decided not to introduce new, specific regulations for nanomaterials, instead assuming that existing frameworks are sufficient, which may not account for the unique complexities of biogenic materials [136]. These legacy frameworks were designed for conventional chemical or biologic products and do not adequately capture the hybrid physicochemical and biological characteristics of mycogenic NPs. Unlike chemically synthesized nanomaterials, fungal-derived NPs are produced through intracellular or extracellular enzymatic processes, resulting in inherently complex surface chemistries, biomolecular coronas, and biological variability. This regulatory gap creates uncertainty in safety evaluation, quality control, and approval pathways.
• Barrier 2—Unclear Classification (Biologic? Drug? Medical Device?): Regulatory classification determines the entirety of a product’s development programme, yet no clear classification pathway exists for mycogenic NPs. Depending on their intended use, composition, and mechanism of action, these materials may be categorized as biologics, drugs, medical devices, or combination products. Regulatory definitions also differ internationally. For example, the European Commission defines nanomaterials using a number-based threshold, typically requiring that 50% or more of particles fall within the nanoscale range of 1–100 nm. In contrast, regulatory approaches in the United States often incorporate mass-based or functional criteria [138]. Fungal-derived nanostructures, especially those incorporating fungal proteins, polysaccharides, or nucleic acids, further complicate classification because they exhibit properties of both biological therapeutics and engineered nanomaterials. This ambiguity complicates regulatory submissions and delays translational development.
• Barrier 3—Lack of Standardised Characterisation Protocols: A major limitation in the regulatory evaluation of mycogenic NPs is the absence of standardized characterization protocols [21]. NP properties such as size, morphology, surface charge, crystallinity, aggregation state, and biomolecular coating depend strongly on fungal species, culture conditions, and synthesis parameters. Currently, measurement procedures vary significantly across laboratories, leading to inconsistent and non-comparable data [178]. The lack of harmonized characterization standards undermines reproducibility, risk assessment, and quality assurance, and prevents the establishment of universally accepted safety and efficacy benchmarks [136,178].
• Barrier 4—No Consensus on Nomenclature and Reporting: The absence of standardized nomenclature and reporting conventions further limits scientific and regulatory progress. Mycogenic NPs are often described using inconsistent terminology (for example: green-synthesised, mycosynthesised, biogenic, fungal-mediated, mycogenic, or ecofriendly), incomplete physicochemical characterization, and non-standard experimental conditions. This lack of reporting uniformity complicates cross-study comparisons, meta-analysis, and regulatory review. Initiatives such as the Minimum Information Reporting in Bio-Nano Experimental Literature (MIRIBEL) framework [179] have emerged to address these challenges by promoting standardized reporting of synthesis conditions, physicochemical properties, and biological interactions. However, adoption remains incomplete, and consensus terminology specific to fungal-derived nanomaterials is still evolving.
• Barrier 5—Good Manufacturing Practice (GMP) Guidelines Unavailable: GMP compliance is mandatory for any clinical-grade pharmaceutical product and covers areas like facility design, personnel qualification, process validation, in-process controls, quality control testing, documentation, and change management. However, the translation of Fungal NP synthesis from laboratory-scale production to industrial-scale manufacturing presents substantial challenges in meeting these requirements. In biogenic nanomaterials, the manufacturing process itself directly determines particle properties, including size distribution, surface chemistry, and biological functionality [138]. Moreover, variations in fungal strain, growth media, incubation time, and purification processes can result in batch-to-batch variability [15], compromising the product consistency and reproducibility that GMP frameworks are specifically designed to ensure Establishing reproducible, standardized manufacturing processes that comply with GMP requirements is essential but remains difficult [136], representing a significant bottleneck in the clinical and commercial advancement of these nanomaterials.
• Barrier 6—Intellectual Property Complexities: Mycogenic NP development faces significant intellectual property and regulatory challenges due to fragmented standardisation, patent uncertainty, and international legal requirements. Parallel standardisation efforts by organisations such as the International Organization for Standardization (ISO), American Society for Testing and Materials (ASTM), and the Organisation for Economic Co-operation and Development (OECD) have resulted in inconsistent technical standards and regulatory expectations, slowing harmonisation and commercial translation [136,138]. Patent protection is also uncertain because fungal NP synthesis relies on natural biological processes, which may be difficult to patent under “product of nature” principles recognised by the United States Patent and Trademark Office. Additionally, international agreements such as the Nagoya Protocol impose legal obligations related to access and benefit-sharing for fungal genetic resources, creating compliance requirements and potential legal risks [180].
• Barrier 7—International Regulatory Harmonisation Lacking: Global regulatory inconsistency represents a significant barrier to commercialization and clinical deployment. Differences in nanomaterial definitions, characterization requirements, reporting metrics, and safety evaluation frameworks across jurisdictions create substantial regulatory uncertainty. For instance, some regulatory bodies prioritize particle number concentration, while others rely on mass concentration or surface area metrics [138]. These differences complicate global approval strategies for fungal-mediated nanomaterials and increase the cost and complexity of regulatory compliance.

### 9.3. Toward Standardisation

Overcoming the regulatory and standardisation deficit for mycogenic NPs requires coordinated action across four domains: reference material development, standard operating procedures, reporting norms, and quality frameworks. These domains are widely recognised as foundational elements for ensuring reproducibility, comparability, and regulatory acceptance of nanomaterials, particularly in medical and biomedical applications.

#### 9.3.1. Need for Reference Materials

The absence of certified reference materials (CRMs) for biologically synthesized nanomaterials, particularly fungal-derived NPs, represents a critical barrier to method validation, interlaboratory comparability, and regulatory acceptance. CRMs provide traceable benchmark values for key physicochemical properties, enabling laboratories to validate analytical methods, assess measurement uncertainty, and ensure consistency of results across institutions and regulatory jurisdictions [138]. Without such standards, accurate verification of instrument performance and measurement reliability is not possible, limiting regulatory confidence and slowing technological translation [138].

Although several organisations, including the US National Institute of Standards and Technology (NIST) and the European Commission Joint Research Centre (JRC), have developed certified nanomaterial reference materials for selected engineered NPs (e.g., NIST SRM 8011/8012/8013 for gold NPs [181]. These materials remain limited in scope and typically address only a small number of physicochemical properties, primarily particle size [136], and do not account for the complex surface chemistry, biomolecular capping, and compositional variability inherent to biologically synthesised nanomaterials.

Furthermore, significant gaps remain in the availability of reference materials suitable for validating characterization methods across diverse nanomaterial classes and applications [138]. The lack of appropriate reference materials has been identified as a major limitation in achieving reliable toxicological evaluation, interlaboratory reproducibility, and regulatory approval [136,182]. The development of CRMs specifically for fungal-derived NPs is therefore essential to establish measurement traceability, support standardized characterization, and enable regulatory approval and industrial translation of these biologically synthesized nanomaterials.

#### 9.3.2. Standard Operating Procedures

Standard operating procedures (SOPs) are essential for ensuring reproducibility and comparability of nanomaterial synthesis, purification, characterization, and biological testing [124]. Unlike chemically synthesised nanomaterials, FNP production involves complex biological systems in which NP formation is influenced by fungal metabolism, enzymatic activity, growth conditions, and extracellular biomolecules [15,66,183]. This biological complexity introduces substantial variability in NP physicochemical properties, including size, morphology, surface chemistry, and functional activity [66]. Reliable risk assessment, regulatory evaluation, and technological translation therefore depend on validated analytical methods supported by well-characterised materials and harmonised protocols [136], specifically tailored to biologically synthesised NPs [66]. However, current nanomaterial research is characterised by significant variability in experimental procedures, and substantial gaps remain in the harmonisation of methodologies and the implementation of standardised operating procedures [138].

ISO, ASTM, and OECD are actively developing consensus-based standards for nanomaterial characterisation, safety assessment, and regulatory evaluation [136,178]. These standards require extensive interlaboratory validation and stakeholder consensus to ensure reliability and regulatory acceptance [178]. However, most existing standards have been developed for chemically synthesised nanomaterials and do not adequately address the unique biological variability, biomolecular surface coatings, and batch-to-batch heterogeneity associated with Fungal NP synthesis. Nevertheless, the absence of harmonised procedures continues to limit the reproducibility and comparability of nanomaterial research and complicates regulatory evaluation [136].

For mycogenic NPs, SOPs must encompass the entire production and evaluation workflow, including fungal strain authentication, standardised culture and synthesis conditions, controlled metal precursor concentration, defined incubation parameters, and reproducible harvesting protocols [66]. In addition, purification procedures must be standardised to ensure removal of residual biomass, extracellular proteins, and metabolic by-products that may influence NP properties and biological responses [66]. SOPs must also define NP dispersion protocols, storage conditions, and contamination screening to ensure stability and consistency during characterisation and biological testing [183]. Implementation of comprehensive SOPs tailored to FNP synthesis is essential to minimise biological variability, ensure reproducibility across laboratories and production batches, and establish the reliability required for regulatory approval and industrial application [66,183].

#### 9.3.3. Reporting Standards and Data Quality Frameworks

Inadequate reporting of synthesis, characterisation, and biological evaluation remains a major obstacle to reproducibility, data comparability, and regulatory assessment of mycogenic NPs. Unlike chemically synthesised nanomaterials, mycogenic NPsare produced through biologically complex processes involving enzymatic reduction, metabolite secretion, and biomolecular capping, all of which significantly influence NP physicochemical properties and biological interactions. However, many studies fail to provide comprehensive characterisation data, including detailed information on particle size distribution, morphology, surface chemistry, and biomolecular composition, preventing meaningful comparison across studies and undermining confidence in safety and toxicity assessments [138]. Incomplete reporting of experimental conditions, characterization methods, and physicochemical properties contributes to uncertainty in risk assessment and limits the usefulness of published data for regulatory decision-making [136].

The availability of complete, high-quality, and standardised reporting is essential for establishing reliable relationships between physicochemical characteristics and biological effects of mycogenic NPs. Harmonised reporting standards and metadata requirements are necessary to ensure that data are complete, traceable, and suitable for regulatory use [138]. Such reporting frameworks facilitate interlaboratory comparisons, enable data reuse, and support the development of predictive models and regulatory risk assessment methodologies.

The MIRIBEL framework, proposed by Faria et al. (2018) [179], established a minimum reporting standard for nanobiology research structured around three core categories: material characterisation, biological characterisation, and experimental protocol details. It provides a valuable foundation for reporting standardisation, improving transparency and reproducibility in nanomaterial research. However, its application to fungal mediated NP systems requires additional biologically specific reporting elements, including complete fungal species and strain identification, culture and synthesis conditions, precursor concentrations, purification procedures, and assessment of potential biological contaminants such as residual proteins, endotoxins, or secondary metabolites. Adoption of the framework remains incomplete, and consensus terminology specific to mycogenic NPs continues to evolve. Establishing and mandating mycogenic NP-tailored reporting requirements would significantly strengthen regulatory confidence and facilitate translational applications.

Regulatory approval of nanomaterial-based products requires rigorous quality assurance to ensure that materials possess well-defined physicochemical properties, consistent performance, and acceptable safety profiles [138]. These requirements are particularly critical for fungal-derived NPs, whose properties are inherently influenced by biological synthesis processes involving enzymatic reduction, metabolite interactions, and biomolecular capping. Reliable and comprehensive characterisation is therefore essential to establish NP size, morphology, surface chemistry, and compositional stability, which directly affect biological activity, functionality, and safety [138].

Implementation of Quality-by-Design (QbD) principles provides a systematic framework for transforming mycogenic NP synthesis into a robust, controlled, and reproducible manufacturing process. QbD emphasises prospective process understanding through identification of critical quality attributes, evaluation of process variability, and establishment of controlled operating ranges to ensure consistent product quality [138]. Application of QbD to fungal NP production would enable optimisation of synthesis parameters such as culture conditions, precursor concentration, and purification processes to ensure reproducible NP characteristics across production batches [15]. Complementary frameworks such as Safe-and-Sustainable-by-Design (SSbD) extend this approach across the full product lifecycle, from synthesis through to waste and reuse, while simultaneously supporting regulatory preparedness by ensuring that oversight mechanisms are developed in parallel with emerging technologies [136,138]. Collectively, these frameworks reposition fungal mediated NP synthesis from a biologically empirical process to a controlled manufacturing system capable of meeting pharmaceutical regulatory requirements, thereby facilitating safe, reproducible, and scalable clinical translation.

## 10. The Knowledge Infrastructure Gap

### 10.1. Current Limitations in Knowledge Integration

The field of mycogenic nanomaterials faces knowledge-transfer issues that impede research and commercialization. Many scientific publications lack critical details in materials and methods, causing researchers to repeat trial and error unnecessarily [184]. This problem is compounded by intentionally created knowledge gaps for intellectual property protection, further fragmenting the available knowledge base [184]. Cross-disciplinary communication is a barrier, as advances in fungal biotechnology often do not reach engineers, architects, and other practitioners. When it does, it remains difficult for non-specialists to interpret, hindering practical implementation. This gap restricts open-source practices needed to accelerate the production and scaling of mycogenic materials [163]. Infrastructure limitations hinder data management and process optimization in fungal research due to unsearchable databases and poor genome annotation quality, complicating data comparison [164]. Fungal biotechnological processes involve long cycles, contamination risks, and multi-step manufacturing, requiring specialized knowledge and infrastructure often unavailable for industrial scale [171]. The field lacks universal platform organisms for material production, as no filamentous fungal chassis exists for standardized heterologous pathway development [164]. The complexity of fungal biology, including multiple nuclei, tough cell walls, and unclear gene expression, leads to unpredictable genetic outcomes [164]. Standardized datasets and protocols could enable deep learning for rational metabolic engineering [164,185].

### 10.2. Digital and Computational Opportunities and Barriers

The integration of AI and automation into fungal cultivation processes presents significant opportunities to revolutionize production efficiency and scalability in mycogenic nanomaterials. AI-driven algorithms can optimize growth conditions in real-time by analysing data from environmental sensors, ensuring that critical parameters such as temperature, humidity, and nutrient concentrations are maintained at optimal levels for NP synthesis [164]. This represents a major advancement over traditional cultivation methods that rely on static protocols and manual monitoring. ML models can predict growth and yield from historical data, allowing proactive cultivation adjustments [164]. This predictive ability could reduce contamination risks and variability, supporting scaling of fungal-based nanomaterials synthesis. Deep learning could enable metabolic engineering by analyzing fungal data to design modifications that increase material production in filamentous fungi [164,185]. Modern biofoundries showcase rapid development cycles, reaching industrial titers in under 90 days [185]. The future of computational integration in mycogenic nanomaterials involves fully automated smart biomanufacturing systems with in silico planning, device connectivity, virtualization, and cloud tools [185]. Automated worklists and ML could enable adaptability and rapid design changes to handle complex fungal biotechnologies. Infrastructure issues hinder data management and analysis, with fungal research lacking the infrastructure for large datasets and facing challenges in data comparison due to inconsistent genome annotation [164]. These gaps hinder essential ML databases. No filamentous fungal chassis is a universal platform because of complex biology, including multiple nuclei, tough cell walls, and unclear gene regulation [164]. These complexities cause unpredictable genetic modifications.

## 11. Cross-Cutting Barriers and Systemic Issues

### 11.1. Funding and Research Ecosystem

The funding landscape for mycogenic nanomaterials research requires strategic investment across multiple levels and sectors. Policy-makers should allocate funding to support research and development initiatives in material science and engineering, prioritizing grants, incentives, and programs that encourage innovation, interdisciplinary collaboration, and the exploration of novel materials [186]. There is a particular need for more publicly funded research at different levels, encompassing the production of mycelium materials to the consumer experience, and identifying any negative or harmful elements or processes that could impact the environment or human health [187]. The U.S. National Nanotechnology Initiative (NNI) launched in 2000 provides a framework for funding nanomaterials research, focusing on synthesis, characterization, education, and application. The revised NNI published in 2011 expanded its focus to include societal and ethical aspects such as “Safe and sustainable development” and “Societal benefits,” while the latest NNI Strategic Plan 2021 emphasizes promoting the “commercialization of nanotechnology” and the “responsible development of nanotechnology” [188]. This model demonstrates how funding initiatives can evolve to address the comprehensive needs of emerging nanotechnology fields, including mycogenic nanomaterials research. The ecosystem must also foster partnerships between universities and industries to bridge the gap between academia and practical applications [186]. Various scientific communication formats and active participation structures for citizens, artists, and designers are of utmost importance for jointly discussing scenarios for a future life and living with and through fungi [189,190]. The research community has identified significant potential in fungal polymers such as chitin and chitosan, which can be harnessed as nanomaterials for medical, pharmaceutical, cosmetic, paper, plastic, and textile applications [190,191].

### 11.2. Education and Workforce Development

Education and workforce development represent critical components for advancing mycogenic nanomaterials research. Policy-makers should invest in educational programs that nurture the next generation of material scientists and engineers, developing partnerships between universities and industries to bridge the gap between academia and practical applications [186]. These educational initiatives must emphasize interdisciplinary collaboration and the exploration of novel materials to prepare students for the complex challenges of working with biological systems and nanotechnology. The U.S. National Nanotechnology Initiative provides a model for integrating education into comprehensive research frameworks. Since its launch in 2000, the NNI has focused on the synthesis, characterization, education, and application of nanomaterials [188]. This demonstrates how educational components can be systematically incorporated into national research strategies for emerging technologies, such as mycogenic nanomaterials. Educational programs must also address ethical considerations related to emerging materials, including their impact on society, health, and privacy, as thoughtful integration of ethical discussions into research can inform responsible material development [186]. This approach ensures that the workforce developing mycogenic nanomaterials is equipped not only with technical skills but also with the ethical framework needed to guide responsible innovation in this emerging field.

### 11.3. Societal and Ethical Dimensions

The societal and ethical aspects of mycogenic nanomaterials require proactive society engagement and strong regulations. Research should include ongoing dialogue, discussing fungal-based materials and their sustainable integration into society [190]. This engagement requires various scientific communication formats and active participation structures for citizens, artists, and designers to jointly discuss scenarios for a future life and living with and through fungi [190]. From a regulatory perspective, robust frameworks must address safety and environmental concerns before widespread commercialization. Regulatory bodies must address the need for robust toxicity profiling and risk assessment frameworks before widespread commercialization [167,192,193]. Policy-makers need to consider ethical issues of new materials, including societal, health, and privacy impacts, integrating these into research for responsible development [186]. Frameworks like the U.S. National Nanotechnology Initiative emphasize expanding policy to include societal and ethical concerns like ‘safe and sustainable development’ and ‘societal benefits.’ Recent plans focus on commercialization and ‘responsible development of nanotechnology’ [188]. A transdisciplinary approach, borrowing from social sciences such as participation and acceptance research, is crucial to explore these materials and educate the public, especially about novel fungal-based materials [189].

## 12. Breaking Through the Barriers: A Roadmap Forward

### 12.1. Immediate Priorities (1–3 Years), Medium-Term Goals (3–7 Years) and Long-Term Vision (7–15 Years)

**Figure 4:**
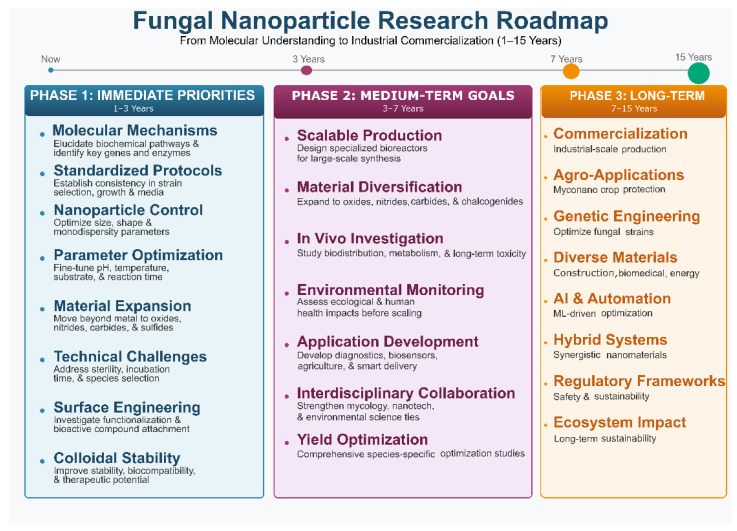
Roadmap for advancing mycogenic nanomaterials from laboratory research to industrial commercialization, highlighting short-term (1–3 years), medium-term (3–7 years), and long-term (7–15 years) priorities, including mechanistic understanding, bioreactor scale-up, synthetic biology integration, AI-driven optimization, and regulatory framework development.

In the short term (1–3 years), the focus should be on elucidating the biochemical and genetic mechanisms governing fungal NP synthesis [27,167], developing standardized and reproducible synthesis protocols [176,194], and achieving precise control over NP size, shape, and surface functionality through the optimization of growth and reaction parameters [27,119]. Expanding synthesis beyond metal NPs and improving understanding of surface biofunctionalization are also essential for enhanced stability and application potential [27]. In the medium term (3–7 years), research should prioritize scalable production using optimized bioreactor systems [14], expansion toward oxide and chalcogenide NPs [48], and a comprehensive evaluation of in vivo behavior and environmental impacts [151,168]. In the long term (7–15 years), integration of synthetic biology, genetic engineering, AI-driven cultivation, and hybrid nanomaterial systems will enable commercialization across agriculture, diagnostics, energy, and biomedicine, supported by strong interdisciplinary collaboration and regulatory frameworks to ensure safety and sustainability [14,164,169,195]. Figure 4 presents the conceptual framework for enabling infrastructure required to transition mycogenic nanomaterials from laboratory-scale synthesis to industrial production.

### 12.2. Enabling Infrastructure

The development of mycogenic nanomaterials relies on specialized bioreactors that can maintain consistent growth conditions, optimize substrate use, and improve production to increase yields [14]. These systems must bridge the gap between lab research and industrial application, requiring robust bioprocess optimization [170,176]. Implementing efficient downstream processing is also essential for enhancing NP quality and yield, making large-scale production more feasible and cost-effective [14]. The integration of AI and automation is a key infrastructure need. AI algorithms optimize growth conditions in real-time by analyzing sensor data for parameters like temperature, humidity, and nutrients [164]. ML predicts growth and yield outcomes, enabling proactive cultivation adjustments. Automating substrate prep, inoculation, and monitoring improves reproducibility and makes fungal products more competitive [164]. Comprehensive monitoring and quality control are vital for managing biological variability. Developing precise, efficient systems is crucial for consistent NP production, especially in the industry where factors like growth conditions, nutrients, and metabolism vary, causing output inconsistencies [161]. Collaboration among microbiology, mycology, nanotech, medicine, and regulation experts is key to translating mushroom-based nanotech from lab to market as a safe, effective tool [168]. Moreover, joint efforts involving synthetic biology, bioprocessing, industry, and regulators are crucial for establishing scalable, quality, and sustainable production frameworks [164]. Establishing standardized regulatory frameworks is a crucial infrastructure need, requiring standardization in synthesis, safety evaluations, and scalable production strategies [169]. These frameworks should consider safety and environmental assessments to guide future research, address constraints, and unlock fungi’s potential in various applications [14].

## 13. Conclusions

Mycogenic nanotechnology represents a promising and innovative approach in modern materials science, where fungi function as sophisticated biological platforms for NP synthesis. Their enzymatic systems, metabolic flexibility, and diverse secretomes enable the production of NPs with natural biological coronas that enhance stability, biocompatibility, and functional activity, making them suitable for applications in medicine, environmental remediation, and sustainable agriculture. While mycogenic nanomaterials hold considerable promise across these fields, their impact is likely to be overestimated in the short-term, given the substantive challenges that currently limit large-scale adoption. These include an incomplete understanding of fungal biosynthetic mechanisms, limited exploration of fungal diversity, scalability constraints, lack of standardized production protocols, and insufficient regulatory frameworks. In addition, comprehensive safety assessments and long-term toxicological studies remain essential for clinical and environmental applications. Among the challenges identified, understanding the fungal biosynthetic pathways represents the most foundational priority, as controlling nanoparticle properties, scalability, and standardization depends on this molecular knowledge. Equally important is the development of globally harmonized regulatory standards through coordinated engagement of organizations such as ISO, OECD, and WHO, supported by multilateral funding frameworks and open-access biosynthetic databases to strengthen reproducibility, transparency, and inclusive global participation.

Nevertheless, these technical and conceptual barriers are ultimately surmountable with sustained and concerted effort across relevant disciplines, including multiomics research, artificial intelligence-driven optimization, synthetic biology, improved bioreactor systems, and international standardization. With coordinated interdisciplinary collaboration and a principled commitment to responsible innovation, mycogenic nanotechnology holds significant potential to become a transformative and sustainable platform for producing next-generation functional nanomaterials. This outlook underscores a clear call to action for researchers, funding institutions and regulatory bodies to align priorities, strengthen collaborative frameworks, and support sustained, evidence-based development of this emerging field toward safe and meaningful real-world application.

## Figures and Tables

**Table 1:** Comparative overview of fungal species used for mycogenic nanomaterial synthesis, including nanomaterial type, synthesis approach, optimized conditions, applications, mechanistic insights, and key physicochemical characteristics.

Study	Fungal Species	Nanomaterial Type	Source of Isolation	Synthesis Approach	Optimized Conditions	Targeted Applications	Mechanism Investigation	Characterization and Features
S. Rajput et al., [108]	*Fusarium oxysporum* (several isolates)	Ag NPs	Not specified	Extracellular	Temperature influenced quantity and particle diameter; pH influenced morphology	Not specified (AgNP properties)	General extracellular biosynthesis by *Fusarium* reported	Colloidal stability dependent on fungal corona (reported)
M. Balakumaran et al., [109]	*Aspergillus terreus* strain PTK 6	Ag and Au NPs	Soil, South India	Extracellular	12 h synthesis (more details in article)	Not specified	Fungal filtrate mediates reduction	Ag: 8–20 nm; Au: 10–50 nm; protein binding confirmed by FTIR
Ahmed M. Abdel-Azeem et al., [110]	*Trichoderma atroviride* (MH283876)	Ag NPs	Endophyte from *Chiliadenus montanus*	Extracellular (culture filtrate)	~25 °C, 72 h biomass; dark reaction; 1 mM AgNO_3_; agitation	Antimicrobial (general)	Biomolecules coat AgNPs	10–15 nm; Ag crystalline peaks; organic layer detected
S. H. Naqvi et al., [111]	*Aspergillus fumigatus*, *A. niger*, *A. flavus*, *A. terreus*	Ag NPs	Not specified	Extracellular (biomass)	~28 °C; 96 h	Comparative AgNP production rates	Species-dependent synthesis efficiency	Size ranges vary; crystalline by XRD
K. Gopinath et al., [112]	*Aspergillus niger*	Cerium oxide (CeO_2_) NPs	Culture collection	Extracellular (culture filtrate)	Growth @ 37 °C; synthesis @ 80 °C; 72 h growth	Antibacterial and larvicidal	Natural enzyme groups act as capping agents	5–20 nm; cubic fluorite structure; SPR ~296 nm
Y. S. Chan et al., [113]	*Pycnoporus sanguineus*	Ag NPs	Not specified	Tested three bioreduction modes	Not specified	Antimicrobial (white-rot fungi)	Fungal filtrate reduction modes tested	52.8–103.3 nm; spherical; SPR ~420 nm
Shreya M. Joshi et al., [114]	*Trichoderma atroviride* strain Tri_AtJSB2	Selenium (Se) NPs	Maintained on PDA	Both extracellular and intracellular	~23 °C; 7 days; 25 mM sodium selenite	Not specified	Biomolecules with –OH/–NH groups involved	60–123 nm; high zeta potential; crystalline
Verma et al., [115]	*Aspergillus clavatus* AzS-275	Ag NPs	Endophytic from *Azadirachta indica*	Extracellular	1 mM AgNO_3_	Not specified	Extracellular reduction	10–25 nm; spherical/hexagonal
H. Mistry et al., [116]	*Aspergillus brunneoviolaceus*	Ag NPs	Marine-derived	Extracellular	36 h study	Not specified	Not reported	~0.7–15 nm; crystalline FCC; SPR ~411 nm
G. Baskar et al., [117]	*Aspergillus terreus*	ZnO NPs	Not specified	Extracellular (filtrate)	Not noted	Antibacterial/antimicrobial	Fungal filtrate influences oxidation	54.8–82.6 nm; FTIR functional groups

**Table 2:** Environmental Remediation Using Mycogenic Nanomaterials.

Mycogenic Nanomaterial	Fungal Source	Pollutant Target	Application	Mechanism	Key Findings	References
TiO_2_ nanoparticles	*Aspergillus penicillioides*	Cd, Cr	Industrial wastewater	Adsorption + photocatalysis	>72% removal of heavy metals; reusable up to 6 cycles	[144]
Iron oxide nanoparticles (IONPs)	*Aspergillus niger*	Cu, Zn, Mn, Cr	Wastewater treatment	Adsorption + magnetic separation	Efficient multi-metal removal from industrial effluent	[145]
Yttrium oxide nanocomposites	*Aspergillus penicillioides*	Pb, Ni	Industrial effluent	Adsorption + catalytic activity	Effective detoxification of toxic metals in wastewater	[146]
Melanin nanoparticles	Filamentous fungi	Heavy metals	Water remediation	Biosorption	Strong heavy metal detoxification via adsorption	[147]
AuNP–mycelial composite	*Aspergillus niger*	Hg^2+^ (mercury)	Water remediation	Adsorption + sensing + reduction	Rapid mercury detection (≤5 µM) and efficient removal	[148]
MnO_2_ biohybrid nanofibers	Mn-oxidizing fungi (*Coprinellus*, etc.)	Heavy metals	Water treatment	Adsorption + ion exchange	Effective multi-metal removal using fungal nanofibers	[149]

## Data Availability

No new data were created or analyzed in this study. Data sharing is not applicable to this article.

## Abbreviations

The following abbreviations are used in this manuscript:

NP	Nanoparticle
ML	Machine-learning
AMR	Antimicrobial resistance
ROS	Reactive oxygen species
CRM	Certified reference material
ASTM	American Society for Testing and Materials
NNI	National Nanotechnology Initiative
QbD	Quality-by-Design
SSbD	Safe-and-Sustainable-by-Design
